# The Use of High-Frequency Skin Ultrasound in the Evaluation of Psoriatic Plaques—A Pilot Comparative Study Between Conventional and Biological Therapy

**DOI:** 10.3390/jimaging12070318

**Published:** 2026-07-13

**Authors:** Adelina Filofteia Ghilencea, Daniel Octavian Costache, Constantin Căruntu, Maria Moga, Raluca Simona Costache

**Affiliations:** 1 Department of Dermatology, Carol Davila University of Medicine and Pharmacy, 010242 Bucharest, Romania; adelina.ghilencea@yahoo.com (A.F.G.); maria.moga@rez.umfcd.ro (M.M.); 2 2nd Dermatology Discipline, Faculty of Medicine, Carol Davila University of Medicine and Pharmacy, 050474 Bucharest, Romania; 3 Physiology Discipline, Carol Davila University of Medicine and Pharmacy, 050474 Bucharest, Romania; costin.caruntu@gmail.com; 4 Internal Medicine and Gastroenterology Department, Carol Davila University of Medicine and Pharmacy, 020021 Bucharest, Romania; 5 The Academy of Romanian Scientists, 050045 Bucharest, Romania

**Keywords:** psoriasis, skin ultrasound, biologic therapy, systemic therapy, PASI score, Doppler, hypoechoic subepidermal band

## Abstract

**Introduction**. Psoriasis is a chronic inflammatory disease histologically characterized by epidermal hyperproliferation, altered keratinocyte differentiation and dermal vascular remodeling. Although the diagnosis is mainly clinical, non-invasive imaging methods, such as high-frequency skin ultrasound, allow an objective assessment of skin changes and disease activity. **Material and Methods**. We conducted a pilot, observational, cross-sectional and comparative study, conducted within the Dermatovenerology Department of the Central Military Emergency Hospital “Dr. Carol Davila”, Bucharest, which included 40 patients diagnosed with psoriasis vulgaris, of whom 22 received conventional systemic treatment (methotrexate 15 mg/week), and 18 received biological therapy. For each patient, a representative, clinically active and recently appeared psoriatic plaque was evaluated with ultrasound, and the thickness of the epidermis, the thickness of the dermis, the thickness of the hypoechoic subepidermal band (SLEB) and the Doppler signal were analyzed. Statistical analysis was performed using SPSS v26. **Results**. Patients under biologic therapy had significantly lower ultrasound parameters compared to those under conventional systemic therapy, especially regarding epidermis thickness, hypoechoic subepidermal band thickness and Doppler signal. The PASI score was significantly higher in the conventionally treated group. Also, significant positive correlations were found between the PASI score and the hypoechoic subepidermal band thickness and the Doppler signal, **Conclusions**. Ultrasound parameters represent useful objective markers in the evaluation of psoriasis, reflecting disease activity. Patients under biologic therapy presented, at the time of evaluation, imaging parameters suggestive of reduced skin inflammation compared to those treated conventionally.

## 1. Introduction

Psoriasis is a chronic inflammatory dermatosis histologically characterized by keratinocyte hyperproliferation, altered epidermal differentiation and dermal vascular remodeling. Although the diagnosis is predominantly clinical, the clinical evaluation of skin lesions may be insufficient if used as a single tool in atypical forms or in situations where an objective assessment of disease activity and therapeutic response is necessary [1]. In this context, non-invasive imaging has become a field of major interest, offering the possibility of the in vivo characterization of skin structures, without compromising the integrity of the skin [2,3,4,5,6,7].

Non-invasive imaging in dermatology includes a set of techniques based on distinct physical principles, such as light reflectance, high-frequency ultrasound, optical interference, or Doppler examination, which allow the visualization of epidermal and dermal structures at different levels of depth and with variable resolutions [2,8,9,10]. Among these, skin ultrasound is a non-invasive, repeatable imaging method based on the reflection of ultrasound waves, the signal variations being determined by the acoustic impedance and the angle of incidence at the interface between tissues [11,12].

In psoriasis, the cardinal ultrasound signs include homogeneous thickening of the epidermal component, visible as a hyperechoic band, the presence of a hypoechoic subepidermal band (subepidermal low-echogenic band—SLEB) and thickening of the dermis, frequently associated with increased Doppler signals in active lesions [13,14,15]. The quantitative parameters commonly used are mainly the thicknesses of the skin structures (epidermis, dermis, total skin depth), the thickness of the SLEB, as well as echogenicity parameters, which have clinical relevance in the assessment of inflammatory activity and therapeutic response [2,12,14,15]. These quantitative ultrasound parameters have increasingly been investigated as potential imaging biomarkers for disease activity and therapeutic response in psoriasis. However, the use of these methods also presents limitations, related to the frequency–resolution–penetration trade-off, the presence of artifacts (e.g., posterior acoustic shadowing in hyperkeratosis or Doppler “blanching” by compression), and the non-specificity of signs such as SLEB [11,13,14].

There is an inverse relationship between frequency and wavelength, with direct implications for penetration and resolution: low frequencies allow for greater penetrability but with reduced resolution, while high frequencies provide superior resolution but with limited depth of examination [11,15]. In dermatology, the need for increased resolution for the assessment of superficial structures has led to the use of frequencies > 15 MHz and the development of high-frequency ultrasound (HFUS) and ultra-high-frequency ultrasound (UHFUS). The latter uses frequencies > 30 MHz and allows for submillimeter resolution of superficial anatomical structures, reaching tens of micrometers for frequencies of approximately 70 MHz, with inherent limitations in depth of penetration [11]. Two main technical configurations are described in the evaluation of psoriasis: dedicated HFUS systems with fixed probes > 20 MHz and high-performance conventional systems, equipped with linear multifrequency probes and sensitive Doppler modules [13,15,16,17]. The ability of Doppler techniques to assess blood flow is of particular importance in assessing the degree of tissue vascularization [11,13,15].

**Figure 1:**
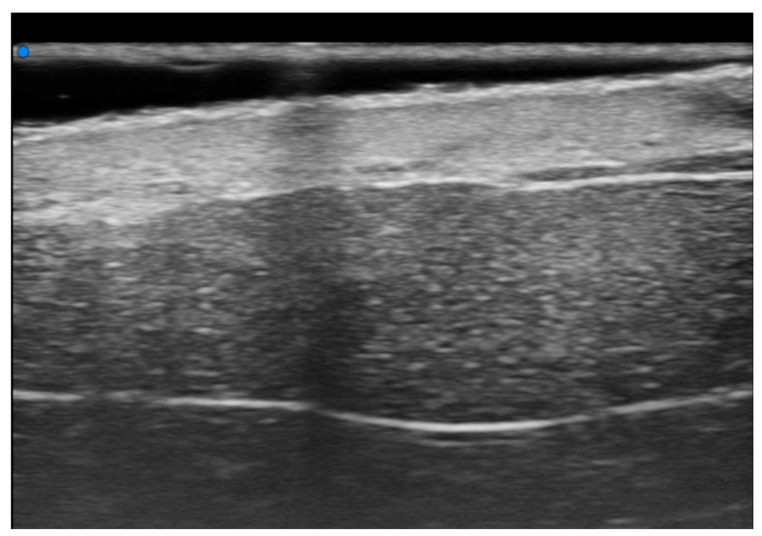
High-frequency ultrasound image of normal skin, showing a thin, continuous hyperechoic epidermal line and a homogeneous dermis with relatively lower echogenicity, without evidence of subepidermal low-echogenic band (SLEB) (UHF 70 MHz).

The ultrasound appearance of normal skin is characterized by a thin, continuous hyperechoic epidermal line and an underlying dermis, which is described as a relatively homogeneous structure, with reduced echogenicity compared with that of the epidermis (Figure 1). Doppler examination plays a key role in assessing dermal vascularization and inflammatory activity. In active lesions, power Doppler allows the detection of increased dermal blood flow, reflecting the degree of local inflammation, but the interpretation of these signals requires the rigorous control of artifacts and the standardization of the examination [13]. Power Doppler is considered more sensitive than color Doppler for detecting low-velocity blood flow within inflamed dermal tissue.

Overall, cutaneous ultrasound is considered a non-invasive, repeatable method capable of providing objective measurements, useful in the longitudinal monitoring of disease and therapeutic response [2,11]. However, it is necessary to deepen the role of skin ultrasound in the evaluation of psoriasis, including determining the possible differences between the various therapeutic options. The aim of this pilot study was to compare the ultrasound characteristics of representative psoriatic plaques in patients receiving biologic therapy or conventional systemic therapy and to explore the relationship between ultrasound parameters and clinical disease severity.

## 2. Materials and Methods

This was a pilot, observational, cross-sectional, comparative, single-center study conducted at the Central Military Emergency University Hospital Dr. Carol Davila, Bucharest, Romania, approved by the local Ethics Committee (approval no. 750/22 January 2025), that aimed to evaluate the ultrasound parameters of psoriatic plaques in patients undergoing conventional systemic treatment compared to those undergoing biologic therapy. This study included 40 patients diagnosed with psoriasis vulgaris, of whom 22 patients were currently on conventional systemic treatment (methotrexate, 15 mg/week), and 18 patients were on biologic therapy—anti-tumor necrosis factor alpha (anti-TNF-α), anti-interleukin 17 (anti-IL-17) and anti-interleukin 23 (anti-IL-23), evaluated at the time of presentation. All biological therapies were analyzed as a single group because it was not possible to perform separate statistical analyses for each class of biological agents due to the small number of patients in each therapeutic subgroup.

The inclusion criteria were age over 18 years, clinical and histological diagnosis of psoriasis vulgaris and the presence of ongoing systemic treatment at the time of evaluation. All patients signed the informed consent. Patients who had recently applied topical treatment to the assessed lesion, those with lesions located in areas difficult to standardize using ultrasound, local skin infection, those with other coexisting inflammatory dermatoses in the examined area, as well as patients with incomplete clinical data were excluded.

As this was an exploratory pilot study, no formal sample size calculation was performed. The number of patients included was determined by the availability of eligible patients during the study period. Demographic, clinical and ultrasound variables were analyzed. Demographic variables included the age and sex of the patients, and clinical variables were the duration of the disease, type of psoriasis, duration of current treatment, type of treatment, Psoriasis Area and Severity Index (PASI) score and location of the examined lesions. Ultrasound evaluation included determination of the thickness of the epidermis and dermis and of the SLEB, as well as assessment of dermal vascularity by Doppler technique.

Ultrasound evaluation was performed using a FUJIFILM SonoSite ultrasound system equipped with the latest ultrahigh-frequency linear probe designed for the examination of superficial structures. A representative, clinically active, and newly appearing psoriatic plaque was selected for each patient to assess active inflammatory changes. All examinations were performed under standardized conditions by the same experienced examiner, who was blinded to the type of treatment of the patient and the PASI score at the time of examination to reduce the risk of observer bias. Ultrasound assessment included measurement of epidermal thickness, dermis thickness, and subepidermal hypoechoic band (SLEB) thickness. Vascularity assessment was performed using a two-step protocol. Initially, all lesions were qualitatively examined using color Doppler, and, in the case of lesions with vascular signal, power Doppler, a more sensitive method for assessing dermal vascularity, was subsequently performed. All examinations were performed using the same device, the same ultrasound settings, and the same examination protocol throughout this study to reduce operator-dependent variability.

Statistical analysis was performed using SPSS version 26. Continuous variables are expressed as median and interquartile range (IQR), as all showed a non-normal distribution. Categorical variables are expressed as frequencies and percentages. Comparison of variables between the two treatment groups was performed using the Mann–Whitney U test for continuous variables and the Chi-square test for categorical variables. Correlations between ultrasound parameters and PASI score were evaluated using the Spearman correlation coefficient, appropriate for variables with a non-normal distribution. Binary logistic regression was used to explore the independent association between ultrasound parameters and achieving complete remission (PASI 100). The statistical significance threshold was set at *p* < 0.05. Given the exploratory nature of this pilot study, no statistical corrections for multiple comparisons were applied. Therefore, the results should be interpreted in an exploratory context and require confirmation by prospective studies conducted on larger groups.

Ethical considerations: This study was carried out in compliance with ethical principles of the Helsinki declaration and it was approved by the ethics committee of the hospital. All the patients signed the informed consent about processing medical data for scientific research purposes.

## 3. Results

The demographic and clinical characteristics of the patients included in this study are presented in Table 1. The mean age in the group was 49.7 years; the majority of the patients were men at 55%, and the median duration of the disease was of 15 years. All of the patients were under treatment for at least one year, receiving either classic medication or biologic therapy. Also, the vast majority had active lesions—70%, with erythema, desquamation and infiltration. These data are presented in Table 1.

**Figure 2:**
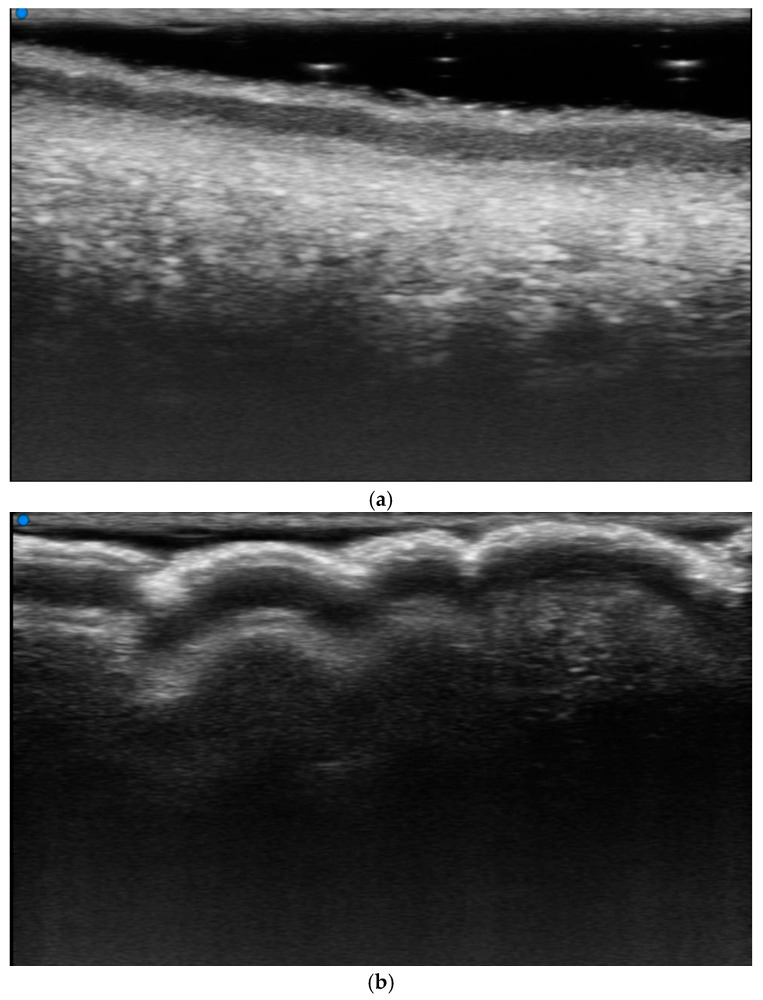
High-frequency ultrasound features of psoriatic plaques: (**a**) subepidermal low-echogenic band (SLEB) associated with dermal thickening, suggestive of active inflammation; (**b**) irregular, undulated epidermal surface corresponding to hyperkeratosis and acanthosis, with underlying dermal changes (UHF 70 MHz).

The following ultrasound parameters were recorded for each patient: epidermal thickness, dermal thickness, Doppler signal and the thickness of the hypoechoic subepidermal band (Figure 2). The median epidermal thickness was 0.4 mm, the median dermal thickness was 2.2 mm, the median hypoechoic subepidermal band was 0.1 mm and the median Doppler signal was 0.5 cm/s. The complete data regarding these parameters are presented in Table 2.

Our main objective was to compare the variables, focusing on ultrasound parameters between those currently under classic therapy with methotrexate and those currently under biologic therapy. In order to achieve this, all the variables between the two subgroups were compared using the Mann–Whitney U test for continuous variables and the Chi-square test for the nominal/ordinal ones. Significant differences were identified, especially regarding the ultrasound parameters—almost all of them were significantly lower under classic therapy with methotrexate than under biologic therapy. In addition, other differences were found in the PASI score—higher in those under classic therapy. For an easier understanding, these aspects are presented in Table 3, and the variables that are significantly different are bolded.

Our next objective was to evaluate the relationship between ultrasound parameters and PASI score using Spearman correlation. The analysis revealed that there was a strong, positive correlation (rs coefficient of almost 0.7 between PASI score and the thickness of the hypoechoic subepidermal band, *p* < 0.001, and with the Doppler signal, *p* < 0.001). In addition, there was a moderate correlation between thr PASI score and thickness of the epidermis—rs coefficient: 0.43, *p*: 0.007—and thickness of the dermis—rs coefficient: 0.40, *p*: 0.01.

Another aim was to explore the relationship between the duration of the treatment and the ultrasound appearance of the lesion. Again, Spearman correlation was used, and it showed that the duration of the treatment negatively correlated with the thickness of the epidermis (rs coefficient 0.35, *p* 0.02), and with the thickness of the dermis (rs coefficient—0.35, *p*—0.02). On the other hand, the other ultrasound parameters did not correlate with the duration of the treatment.

Lastly, we tried to identify the ultrasound parameters that best correlated with the good clinical control of the disease. Firstly, we explored the differences in ultrasound parameters between those who were in complete remission at the moment of evaluation, which meant PASI 100, and those who were not using the Mann–Whitney U test. The results were very promising, with almost all of the parameters being significantly lower in those who achieved PASI 100—Table 4.

Secondly, we applied the Spearman correlation test to evaluate if there were significant correlations between the ultrasound parameters in order to build a binary logistic regression model. The only significant correlation, with rs over |0.7|, was between epidermal thickness and the hypoechoic subepidermal band thickness. Lastly, in the logistic regression, we introduced epidermal thickness and Doppler signal as independent variables and, as an independent variable, PASI 100. However, despite a Nagelkerke R2 of 0.69, Chi-square of 25 and *p* < 0.001, which demonstrate a very good overall model, none of the variables were independently associated with PASI 100. Most probably, the explanation resides in the relatively small number of patients in our cohort.

## 4. Discussion

This pilot study highlights that high-frequency cutaneous ultrasound can serve as an objective, non-invasive tool for assessing disease activity in patients with psoriasis vulgaris. The evaluated ultrasound parameters correlated with clinical severity as measured by the PASI score, supporting the role of ultrasound as complementary to clinical examination in assessing cutaneous inflammation and treatment response.

The data from the literature show that ultrasound changes, including reductions in the thickness of the epidermis and dermis and in the SLEB, occur early under treatment and can be used to monitor the therapeutic response [15,18].

The results are consistent with the current literature, which highlights the usefulness of high-frequency ultrasound in the evaluation of psoriasis, both for the morphological characterization of lesions and for monitoring the response to treatment. Ultrasound allows the identification of characteristic changes in psoriatic plaques, such as thickening of the epidermis, the presence of a low-echogenicity subepidermal band and increased dermal vascularization, providing objective parameters [18,19,20,21]. From a clinical point of view, the results of this study support the integration of skin ultrasound as a complementary method in the evaluation of psoriasis due to its non-invasive, repeatable nature and its ability to detect even subclinical changes. Ultrasound is also considered a useful method for the early identification of inflammation and for monitoring the evolution of the disease [16,20,21,22]. Moreover, recent studies suggest that ultrasound can also detect subclinical inflammation even in patients considered to be in clinical remission, highlighting its role as a sensitive and valuable assessment tool [16,21,23].

However, the differences observed between the two treatment subgroups should be interpreted with caution. Patients receiving biologic therapy had a significantly longer duration of both disease and treatment compared to those receiving conventional therapy, reflecting the usual therapeutic choice of patients with moderate-to-severe psoriasis. Therefore, the groups were not comparable in terms of baseline characteristics, and the observed ultrasound differences cannot be attributed solely to the type of treatment administered. In this context, this study suggests that ultrasound is able to reflect the inflammatory status of the patient at the time of examination and highlight objective differences in disease activity. From a clinical perspective, assessing SLEB thickness and the Doppler signal can provide additional information beyond the clinical examination, contributing to an objective assessment of inflammatory activity and therapeutic response. Cutaneous ultrasound can be particularly useful in identifying residual or subclinical inflammation, even in cases where lesions show apparent clinical improvement [18,19,23,24].

Although the logistic regression model showed a satisfactory overall performance, none of the ultrasound parameters remained independently associated with achieving PASI100. This result can be explained both by the relatively small number of patients who achieved complete remission and by the existing correlation between the ultrasound parameters analyzed, which reflect different manifestations of the same inflammatory process. Therefore, these results should be interpreted with caution and validated in larger cohorts.

The present study has several limitations that must be taken into consideration when interpreting the results. Firstly, as it is a pilot, single-center study conducted on a relatively small number of patients, the results should be interpreted with caution, and their generalizability to the wider population of patients with psoriasis is limited. Secondly, the cross-sectional design only allowed the assessment of ultrasound parameters at a single point in time, without the possibility of establishing a causal relationship between the type of treatment and the observed imaging changes or of evaluating their evolution over time. Thus, the differences identified between the groups reflect the inflammatory status at the time of examination and cannot be interpreted as evidence of the superiority of a therapeutic strategy.

Another limitation is represented by the heterogeneity of the group treated with biologic therapy, which included patients under anti-TNF-α, anti-IL-17 and anti-IL-23 treatment. Due to the small number of patients in each subgroup, it was not possible to perform separate comparative analyses between the different classes of biological therapies. Also, although all ultrasound examinations were performed by the same examiner, using a standardized protocol and the same instrument settings, a formal assessment of intraobserver reproducibility was not performed. In addition, the relatively small number of patients who achieved complete remission (PASI100) may explain the lack of independent associations in the logistic regression analysis.

In the future, prospective, multicenter studies with larger cohorts, repeated ultrasound assessments and standardized protocols are needed to validate skin ultrasound as an imaging biomarker of disease activity and therapeutic response, as well as to compare the effects of different classes of biological therapies on ultrasound parameters.

## 5. Conclusions

This pilot study suggests that high-frequency skin ultrasound is an objective, non-invasive, and reproducible tool for assessing disease activity in patients with psoriasis vulgaris. The ultrasound parameters analyzed, in particular the thickness of the hypoechoic subepidermal band (SLEB) and the Doppler signal, correlated with clinical severity assessed by the PASI score, supporting their role as imaging markers of skin inflammation.

Patients receiving biologic therapy had ultrasound parameters suggestive of a lower degree of inflammation at the time of evaluation compared with patients who were conventionally treated. However, given the cross-sectional design of this study and the differences between groups regarding disease duration and treatment duration, these results should not be interpreted as evidence of the superiority of one therapeutic strategy.

Overall, we support the integration of skin ultrasound as a complementary method to the clinical evaluation and monitoring of patients with psoriasis. Prospective, multicenter studies, conducted on larger cohorts and with serial ultrasound assessments, are needed to validate these results and define the role of ultrasound as an imaging biomarker of disease activity and of therapeutic response.

## Figures and Tables

**Table 1:** General characteristics of patients.

Variable	Results (40 Patients)
Age, mean (SD; CI)	49.7 (12.1; 45.8–53.6)
Sex, M (%)	22 (55%)
Length of disease, years, median (IQR; CI)	15 (13; 12.5–18.8)
Type of treatment, n (%)	Methotrexate—22 (55%) Biologic therapy—18 (45%) • Anti-TNF-α—6 (15%) • Anti-IL-17—7 (17.5%) • Anti-IL-23—5 (12.5%)
Active lesions, n (%)	28 (70%)
Erythema, n (%)	Absent—12 (30%) Low—8 (20%) Moderate—17 (42.5%) Severe—3 (7.5%)
Desquamation, n (%)	Absent—12 (30%) Low—15 (37.5%) Moderate—10 (25%) Severe—3 (7.5%)
Infiltration, n (%)	Absent—12 (30%) Low—17 (42.5%) Moderate– 8 (20%) Severe—3 (7.5%)
PASI score, median (IQR; CI)	2 (4; 1.76–3.89)
PASI 100, n (%)	13 (32.5%)
Affected region, n (%)	Upper limb—11 (27.5%) Lower limb—12 (30%) Trunk—17 (42.5%)

CI—confidence interval; IL-17—interleukin 17; IL-23—interleukin 23; IQR—interquartile range; M—male; n—number; PASI—psoriasis area and severity index score; PASI 100—complete remission at moment of evaluation; SD—standard deviation; TNF-α—tumor necrosis factor alpha; %—percentage.

**Table 2:** Ultrasound parameters.

Variable	Results (40 Patients)
Epidermal thickness, mm, median (IQR; CI)	0.4 (0.3; 0.37–0.49)
Dermal thickness, mm, median (IQR; CI)	2.2 (0.7; 2.13–2.65)
Subepidermal low echogenic band (SLEB) thickness, mm, median (IQR; CI)	0.1 (0.5; 0.19–0.44)
Doppler signal, cm/s, median (IQR; CI)	0.5 (1.2; 0.47–1.09)

CI—confidence interval; cm—centimeters; cm/s—centimeters per second; IQR—interquartile range.

**Table 3:** Comparison of demographic, clinical and ultrasound characteristics according to treatment group.

Variable	Classic Therapy (22 Patients)	Biologic Therapy (18 Patients)	*p*-Value *
Age, mean (SD; CI)	47 (13.6; 40.95–53.05)	53.1 (9.3; 48.47–57.75)	0.89
Sex, M, n (%)	13 (59.1%)	9 (50%)	0.75
**Duration of disease, years, median (IQR; CI)**	10 (15; 7.29–16.17)	20 (11; 16.96–24.15)	0.002
**Duration of current treatment, years, median (IQR; CI)**	1 (0; 0.95–1.14)	3.5 (3; 2.64–6.25)	<0.001
Active lesion, n (%)	18 (81.8%)	10 (55.6%)	0.09
Erythema, n (%)	Absent—4 (18.2%) Low—5 (22.7%) Moderate—11 (50%) Severe—2 (9.1%)	Absent—8 (44.4%) Low—3 (16.75) Moderate—6 (33.3%) Severe—1 (5.6%)	0.14
Desquamation, n (%)	0—4 (18.2%) 1—9 (40.9%) 2—8 (36.4%) 3—1 (4.5%)	Absent—8 (44.4%) Low—6 (33.3%) Moderate—2 (11.1%) Severe—2 (11.1%)	0.14
Infiltration, n (%)	0—4 (18.2%) 1—10 (45.5%) 2—6 (27.3%) 3—2 (9.1%)	0—8 (44.4%) 1—7 (38.9%) 2—2 (11.1%) 3—1 (5.6%)	0.08
**PASI score, median (IQR; CI)**	4 (6; 2.64–6.11)	1.2 (2; 0.5–1.7)	0.002
PASI 100, n (%)	5 (25%)	8 (44%)	0.30
Affected area, n (%)	Upper limb—7 (31.8%) Lower limb—5 (22.7%) Trunk—10 (45.5%)	Upper limb—4 (22.2%) Lower limb—7 (38.9%) Trunk—7 (38.9%)	0.52
**Epidermal thickness, mm, median (IQR; CI)**	0.5 (0.3; 0.41–0.58)	0.3 (0.1; 0.27–0.44)	0.02
Dermal thickness, mm, median (IQR; CI)	2.31 (0.7; 2.20–2.87)	2.1 (0.6; 1.78–2.64)	0.05
**Hypoechoic subepidermal band thickness, mm, median (CI; IQR)**	0.5 (0.6; 0.27–0.59)	0 (0.3; 0.01–0.38)	0.01
**Doppler signal, cm/s, median (IQR; CI)**	1 (1.5; 0.64–1.56)	0 (1; 0.03–0.74)	0.02

CI—confidence interval; cm—centimeter; cm/s—centimeters per second; IQR—interquartile range; M—male; n—number; PASI—psoriasis area and severity index; PASI 100—complete remission at the moment of evaluation; SD—standard deviation; %—percentage; *—considered significant under 0.05.

**Table 4:** Ultrasound parameter differences between those with complete remission and those without.

Variable	Complete Remission	No Complete Remission	*p*-Value *
Epidermal thickness, mm, median (IQR; CI)	0.3 (0.1; 0.22–0.39)	0.45 (0.2; 0.42–0.58)	0.001
Dermal thickness, mm, median (IQR; CI)	2 (0.8; 1.7–2.51)	2.3 (1; 2.18–2.91)	0.08
Hypoechoic subepidermal band thickness, mm, median (IQR; CI)	0 (0; 0–0)	0.5 (0.5; 0.32–0.65)	<0.001
Doppler signal, cm/s, median (IQR; CI)	0 (0; 0–0)	1 (1; 0.8–1.61)	<0.001

CI—confidence interval; cm—centimeter; cm/s—centimeters per second; IQR—interquartile range; *—considered significant under 0.05.

## Data Availability

The raw data supporting the conclusions of this article will be made available by the authors on request.
